# Supplementary material for: Phase Contrast Image-Based Rapid Antimicrobial Susceptibility Testing of Bacteria in Liquid Culture Media
Source: Sensors (Basel). 2022 Dec 21;23(1):59. doi: 10.3390/s23010059 (PMC9824109; doi:10.3390/s23010059)
Supplement: Supplementary file 1 [file sensors-23-00059-s001.zip › sensors-2072462-supplementary.pdf]

Supplementary information:

Phase Contrast Image-Based Rapid Antimicrobial Susceptibility Testing of Bacteria in Liquid Culture Media

Xiaonan Zhang [1,2], Xuenian Wang [2], Yaqing Bao [3], Zhiyuan Shen [4], Yang Xu [2], Bei Wang [2], Haowei Zhang [5], Tian Guan [1,2] and Yonghong He [2,6,*]

[1] School of Medicine, Tsinghua University, Beijing 100084, China
[2] Institute of Optical Imaging and Sensing, Shenzhen Key Laboratory for Minimal Invasive Medical Technologies, Shenzhen International Graduate School, Tsinghua University, Shenzhen 518055, China
[3] GBA Center for Medical Device Evaluation and Inspection, National Medical Products Administration, Shenzhen 518045, China
[4] Medical Optical Technology R&D Center, Research Institute of Tsinghua, Pearl River Delta, Guangzhou, 510700, China
[5] School of Life Sciences, Tsinghua University, Beijing 100084, China
[6] Department of Physics, Tsinghua University, Beijing 100084, China
[*] Authors to whom correspondence should be addressed.

1. Comparison of AST methods

Table S1 shows the comparison of traditional methods, existing rapid antimicrobial susceptibility testing(AST) methods and our method in terms of testing time, cost and complexity.

Table S1. Comparison of AST methods

	Traditional methods	Rapid AST in solid culture media	Rapid AST in liquid culture media	Our method
Examples of techniques	Disk diffusion method, broth dilution method, etc.	Agarose based rapid AST	Microfluidics based rapid AST	Centrifugation-single field of view tracking-image processing
Time of Test	16-24 hours	Within 4 hours	Within 4 hours	4 hours
Cost	Low	High	High	Low
Complexity	Low	High	High	Low

2. Results of centrifugation-single field of view tracking-image processing method at the 24th hour

We photographed the bacterial images of the centrifuged bacterial solution in the counting plate at the 24th hour under the effect of five tigecycline concentrations (Figure S1).

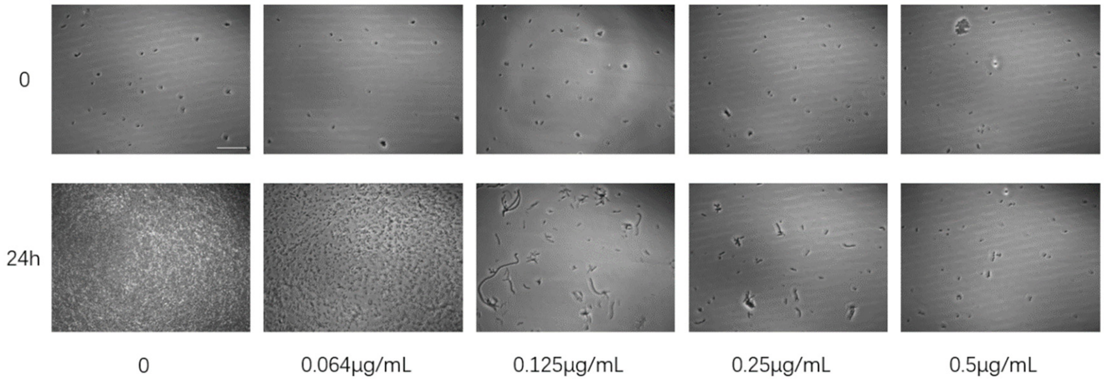

Figure S1. Bacterial images at the 24th hour. The horizontal axis represents the concentration of tigecycline in the bacterial solution, and the vertical axis represents incubation time. Scale bar, 30μm.

As can be seen from the figure, after 24 hours, the drug-free and 0.064 μg/mL concentrations of tigecycline were full of *E. coli* growth, indicating that the 0.064 μg/mL concentration of tigecycline did not inhibit the growth of *E. coli*; in the 0.125 μg/mL and above concentrations, the increase in the number of *E. coli* was not obvious, although in the 0.125 μg/mL tigecycline solution, the *E. coli* morphology increased somewhat, but the number of *E. coli* did not show exponential growth, so the lowest inhibitory concentration detected by our proposed method was still 0.125μg/mL after 24 hours of incubation.

Based on the above images, we calculated the growth rate of *E. coli*(Figure S2), and it can be seen that the growth rate of *E. coli* increased greatly at 0 and 0.064μg/mL concentrations, and did not change much at 0.125μg/mL and above. We can conclude that after 24 hours, the minimum inhibitory concentration detected by our method is still 0.125μg/mL.

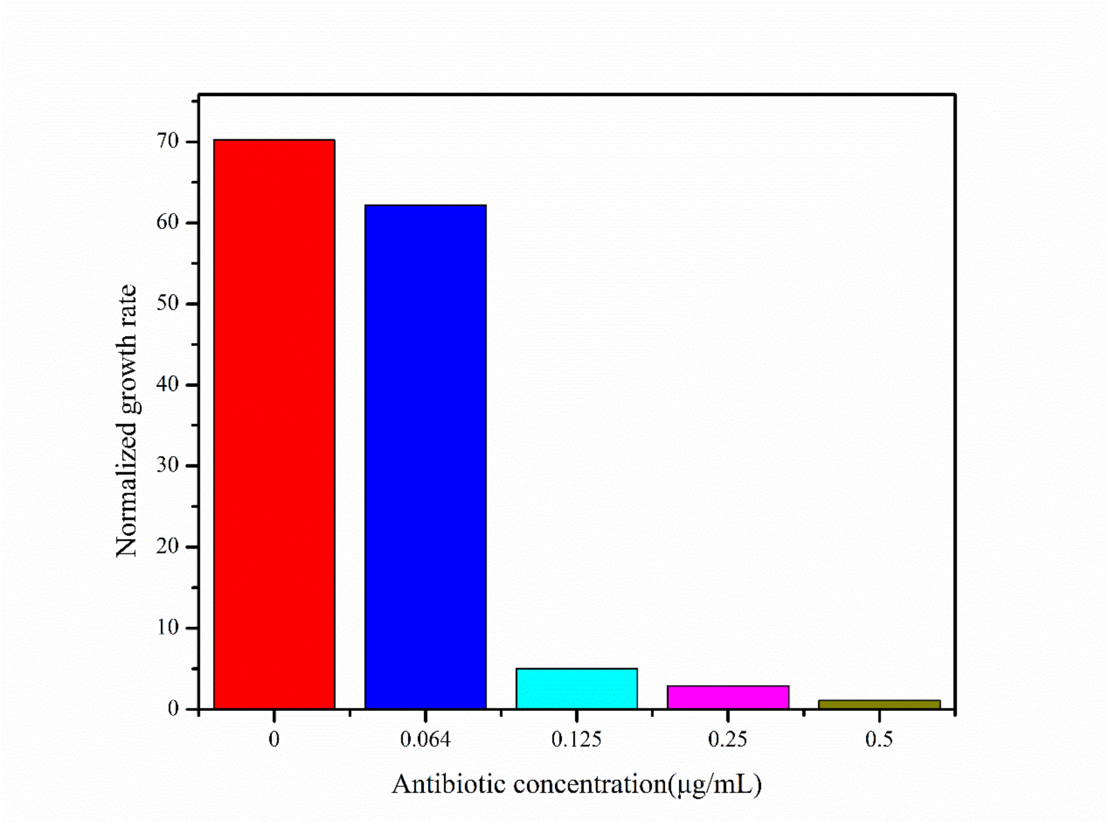

Figure S2. Bacterial growth rate after 24 hours of use centrifugation-single field of view tracking-image processing method
